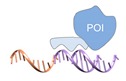# Manipulating Enzymes Properties with DNA Nanostructures

**DOI:** 10.3390/molecules24203694

**Published:** 2019-10-14

**Authors:** Andreas Jaekel, Pierre Stegemann, Barbara Saccà

**Affiliations:** ZMB, University Duisburg-Essen, Universitätstraße 2, 45117 Essen, Germany; andreas.jaekel@uni-due.de (A.J.); pierre.stegemann@uni-due.de (P.S.)

**Keywords:** DNA nanotechnology, DNA-protein conjugates, spatial confinement, entropic avidity, enzymatic assays

## Abstract

Nucleic acids and proteins are two major classes of biopolymers in living systems. Whereas nucleic acids are characterized by robust molecular recognition properties, essential for the reliable storage and transmission of the genetic information, the variability of structures displayed by proteins and their adaptability to the environment make them ideal functional materials. One of the major goals of DNA nanotechnology—and indeed its initial motivation—is to bridge these two worlds in a rational fashion. Combining the predictable base-pairing rule of DNA with chemical conjugation strategies and modern protein engineering methods has enabled the realization of complex DNA-protein architectures with programmable structural features and intriguing functionalities. In this review, we will focus on a special class of biohybrid structures, characterized by one or many enzyme molecules linked to a DNA scaffold with nanometer-scale precision. After an initial survey of the most important methods for coupling DNA oligomers to proteins, we will report the strategies adopted until now for organizing these conjugates in a predictable spatial arrangement. The major focus of this review will be on the consequences of such manipulations on the binding and kinetic properties of single enzymes and enzyme complexes: an interesting aspect of artificial DNA-enzyme hybrids, often reported in the literature, however, not yet entirely understood and whose full comprehension may open the way to new opportunities in protein science.

## 1. Introduction

In 1982, Nadrian Seeman published the design of the first immobile Holliday junction, composed of four distinct strands intertwined at a common crossover point [1]. A few years later, the same motif was used to realize a periodic network of indefinite size through cohesion of complementary sticky-ends protruding from opposite arms of the tile [2]. The idea to use DNA as a construction material demonstrated to be as brilliant as simple, signing the beginning of a new field of science, now known as structural DNA nanotechnology. Almost 40 years of successful works have consolidated the fundamental principles of the field and opened avenues which were previously only hardly imaginable, such that nowadays DNA objects of almost any desired size and shape can be realized in a short time and—most importantly—with spatial control at the nanometer scale level [3]. These efforts culminated in the realization of a macroscopic 3D crystal made entirely by the self-assembly of a rationally designed DNA tile [4], demonstrating the possibility to bridge the nano- to the macroscopic world in a programmable fashion. 

An important step forward was done with the introduction of reconfigurable modules at distinct locations of the DNA nanostructure [5]. These modules are typically small motifs that can exist in (at least) two distinct and well-defined conformational states. Selective switching from one state to the other is driven by changes in ions concentration [6], light irradiation [7], pH [8,9], or DNA itself [10,11,12] and is used to trigger the global mechanical transformation of the structure in a coordinated and often reversible way. Control of matter distribution can be thus applied at different times, enabling—in Seeman’s words—“to put any molecule you want, where you want, when you want it there” [13]. 

**Figure 1:**
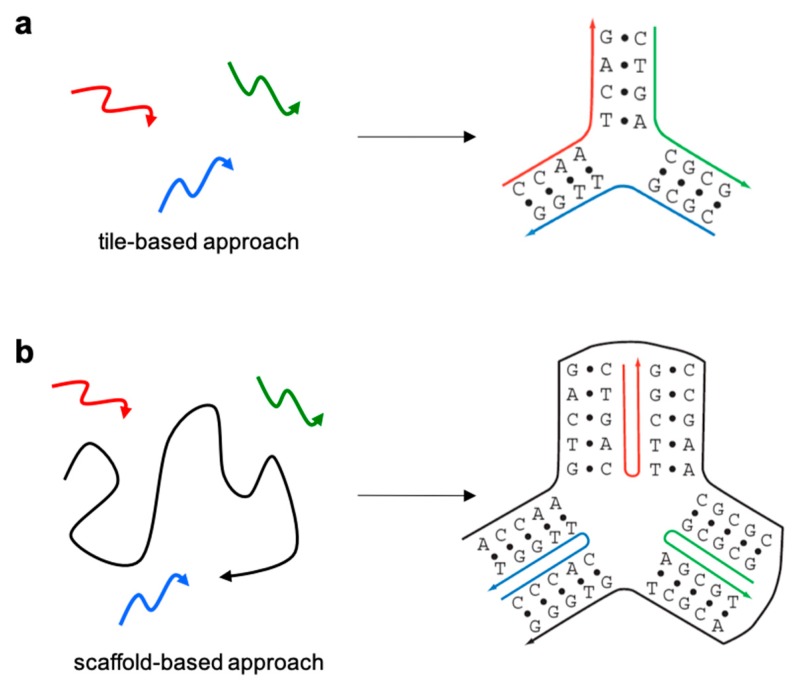
Schematic representation of the two main design strategies currently adopted for the realization of DNA nanostructures: (**a**) the tile-based and (**b**) the scaffold-based (or DNA origami) approach.

Several years after the foundation of DNA nanotechnology, an alternative self-assembling strategy was developed, later called scaffolded DNA origami [14]. The main difference between the two assembly strategies relies on the presence or absence of a long single-stranded DNA (termed scaffold or template) in the reaction mixture (Figure 1). In the tile-based approach, originally developed by Seeman, relatively short oligonucleotides (20–40 bases long) are designed to intertwine one another into branched motifs of defined geometry [15], following a rather strict principle of sequence-symmetry minimization [16]. According to this principle, sequences participating in the formation of the motif are chosen to be maximally different from one another, such that competitive formation of alternative secondary structures is minimized (Figure 1a). Despite careful design efforts, the relatively small size and high structural flexibility of such tiles inevitably result in their partial dissociation at room temperature. A way to escape this drawback is to connect tiles into large periodic lattices through cohesion of their complementary sticky-ends. Alternatively, the intrinsic dynamics of such small DNA motifs can be advantageously used to generate complex reaction networks and DNA circuits, whose kinetic behavior is governed by predictable single-strand displacement mechanisms [5,17,18,19]. A major bottleneck to the successful implementation of this self-assembly strategy is, however, the necessity to achieve a high control over the stoichiometry and purity of the constituent oligonucleotides, resulting in error-prone and lengthy synthetic processes. 

When the goal is instead to realize a robust framework, where structural features can be singularly addressed with a precision of only a few nanometers, the DNA origami method is the most appropriate choice (Figure 1b). Similar to the tile-based approach, the DNA origami method relies on the intertwined connection of Holliday junctions into large patterns. However, in this case, a ca. 7.000 bases-long scaffold is folded into a desired two-dimensional (2D) or three-dimensional (3D) shape through discontinuous hybridization to hundreds of shorter oligonucleotides (also named staple strands). Since staple strands hybridize to a shared sequence rather than with each other, their relative stoichiometric ratio is no more relevant. Moreover, once nucleation starts, the structure rapidly grows, guided along the folding pathway by the formation of progressively more stable intermediates till a minimum of energy is reached [20], with obvious advantages in terms of yield and assembly time. Altogether, the DNA origami method demonstrated to be robust and easy to implement, contributing to the impressively rapid evolution of the field and its spreading among more than 400 laboratories around the world. Thus, starting from a simple four-strands motif, DNA nanotechnology developed to a level of design sophistication and structural reliability that is nowadays suitable for many applications in diverse scientific disciplines, ranging from theoretical and computational biology to nanoplasmonic, nanoscopy, nanomaterials, nanomedicine and synthetic biology (for recent reviews on the topic, see [21,22,23,24,25,26]). 

This review has the purpose to give an overview on that particular aspect of DNA nanotechnology that deals with proteins (particularly enzymes): from the synthesis of DNA-tagged proteins (herein referred to as DNA-protein conjugates) till their integration onto large DNA scaffolds (resulting in herein called DNA-protein nanostructures), with a special focus on their binding affinity and catalytic activity. Clearly, the first fundamental step for the study of such hybrid materials is the attainment of a homogeneous chemical species with a high purity grade, such that any observable change in the biophysical properties of the DNA-protein nanostructure can be unambiguously attributed to a defined structural feature of the sample. We will, therefore, start with an overview of the chemical methods typically adopted for the synthesis of DNA-protein conjugates, their tethering to a DNA scaffold and final purification (Section 2). This section will provide the reader with generic information on the topic and is not intended to be exhaustive. A more extensive treatment of the subject can be found in several other reviews, to which the interested reader is encouraged to refer [26,27,28,29,30,31,32]. Section 3 will instead describe the various geometries adopted by DNA-scaffolded proteins, a structural aspect that should be considered if the binding and catalytic properties of the final construct are supposed to be dependent on the way the chemical space around the protein is modified. The most relevant parts of this review will be centered on the use of DNA nanostructures as scaffolds for the multivalent binding of identical ligands to oligomeric proteins (Section 4) and as enhancers of the catalytic activity of the bound enzyme or enzyme cascades (Section 5). Both aspects will be discussed also in terms of theoretical models that have been proposed to understand the observed phenomena. Many of these hypotheses are still matter of debate in the community and cover various facets of the problem, from the symmetry of the system to the structural conformation of the protein and its allosteric regulation, surface density charge and distance-dependent interactions with or along the DNA component. The existence of different theories and the difficulty to demonstrate all of them experimentally evidences our still limited knowledge of DNA-protein nanostructured systems, whose deeper understanding is not only scientifically stimulating but offers also the opportunity to generate semisynthetic materials with improved and programmable properties. The review will end with general comments on current challenges and future perspectives in the field (Section 6). 

## 2. Chemical Strategies for the Synthesis and Purification of DNA-Protein Conjugates

**Figure 2:**
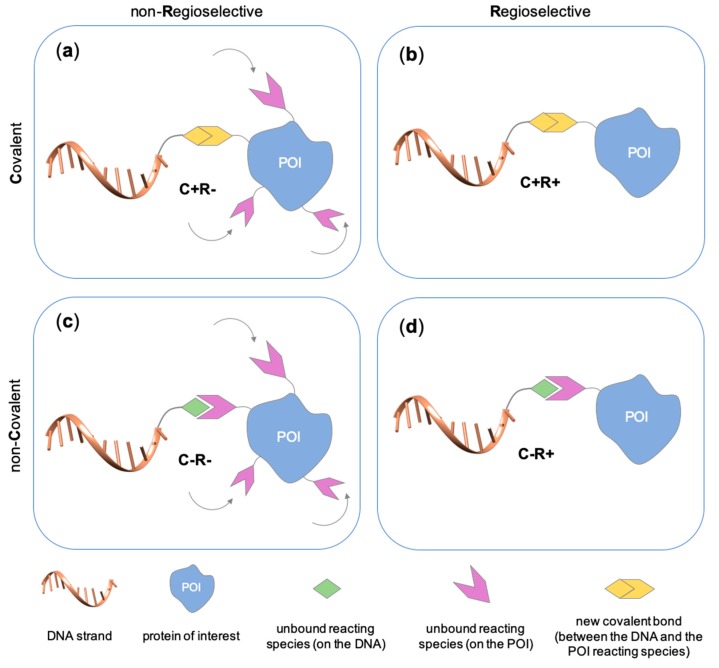
Schematic representation of the chemical methods typically adopted for the synthesis of DNA-protein conjugates. The strategies can be classified according to the establishment of a covalent (C+) or non-covalent (C-) bond between the DNA and protein component as well as on the occurrence (R+) or absence (R-) of regioselectivity. Accordingly, four classes can be distinguished: from (**a**) to (**d**), C+R-, C+R+, C-R- and C-R+. The establishment of a covalent bond between the DNA handle (green symbol) and the chemical moiety (magenta symbol) attached to the protein of interest (POI, in blue) gives rise to a new chemical group between the two molecules (yellow symbols merged together). A non-covalent bond instead preserves the identity of the interacting units (green and magenta symbols for the DNA and the POI handles, respectively).

Several approaches have been developed until now and are in continuous evolution to enable the binding of a protein of interest (POI) to a DNA molecule. The methods used for the synthesis of DNA-protein hybrids typically include chemical conjugation, enzymatic ligation or a combination of both, and are applied either to native proteins or their genetically modified variants. A convenient way to classify these strategies relies on two parameters of the newly established interaction: strength and selectivity. For the strength of the bond, we intend the possibility for the DNA-protein interaction to be either covalent, and thus mainly strong and irreversible, or non-covalent and therefore weaker and amenable to dissociate easily. In addition, the newly formed bond may be directed exclusively to one specific site of the POI (i.e., the DNA-protein interaction is regioselective) or address many sites, which are chemically identical and spatially indistinguishable (i.e., the DNA-protein interaction is non-regioselective). Of course, a pre-requisite of all these approaches is to be chemo-selective, meaning that the reaction under investigation is designed to take place only among specific chemical groups appended respectively to the DNA and protein molecule, although in few cases alternative reaction pathways and outcomes cannot be completely excluded. According to this definition, we can distinguish four classes of DNA-protein conjugation methods (Figure 2), in which the newly established interaction is either covalent (C+) or non-covalent (C-) and, for each of these two scenarios, may or may not display regioselectivity (R+ or R-). The four strategies described in this section refer to the establishment of a “link” between a single-stranded DNA and a POI, giving rise to properly called DNA-protein conjugates. Some of these strategies have been also successfully used for further protein decoration of larger DNA constructs, as described in Section 2.3, resulting in the formation of DNA-protein nanostructures. 

### 2.1. Covalent Methods

We will start our survey with a brief summary of the covalent methods used for the synthesis of DNA-protein conjugates. A major advantage of this method is the formation of a stable bond between the two reactants, although this is often counteracted by the necessity to functionalize the protein with a specific reactive group or a small protein tag prior to DNA conjugation. This normally requires the implementation of a few chemical steps and/or genetic manipulations, followed by purification of the modified protein and final linkage to the DNA handle. Despite extensive procedures may compromise the structural integrity and activity of the protein, reaction conditions and genetic handling have been optimized to minimize these risks, making covalent strategies still one of the methods of choice for the synthesis of DNA-protein conjugates.

#### 2.1.1. Covalent and Non-Regioselective Methods (C+R-)

One of the most largely used and successful approaches for DNA-protein conjugation relies on thiol chemistry. Pioneering works were based on the formation of disulfide bonds between an alkyl thiol-modified DNA and a cysteine residue of *staphylococcus* nuclease [33]. While this method suffers from bond cleavage under reductive conditions, bifunctional heterocross-linkers (such as sulfo sSMMC) enable irreversible coupling. These linkers bear an active ester for amide coupling to the Lys residues exposed on the protein surface and a maleimido moiety for orthogonal binding to a thiol-modified DNA (Table 1). Despite being largely established and normally straightforward to perform, this synthetic approach is accompanied by a major drawback. Indeed, the lack of regioselectivity due to the presence of typically more than one lysine residue on the target protein leads to a heterogeneous mixture of products with an undefined number of DNA chains linked to an unknown combination of accessible amine residues (Figure 2a). In some cases, the desired 1:1 DNA:protein conjugate can be isolated from the distribution of possible products through high-performance liquid chromatography, although careful optimization of purification protocols is necessary and is not always a guaranty of success. In other cases, this problem can be even fully circumvented, enabling the site-selective modification of the protein. Indeed, when the protein displays one single Cys residue, either in its native or genetically engineered form, and this position can be addressed without compromising the stability of the structure, the same thiol chemistry described above can be applied to link the protein to an amino-modified DNA chain (Table 1). 

#### 2.1.2. Covalent and Regioselective Methods (C+R+)

When cysteine engineering is not an option, alternative methods can be employed to express the protein of interest with a chemical handle. This can be further covalently coupled to a specific functional group previously attached to a DNA strand, resulting in the formation of a stable linkage between this latter and the protein (Figure 2b). These techniques require genetic manipulation of the POI to introduce the desired mutation and can be therefore challenging to implement, time consuming and normally need to be optimized for each specific protein. Some representative examples are reported below (Table 2). 

Azido-chemistry. A common approach for site-selective protein labelling is based on azido-alkyne cycloaddition. Accordingly, the POI must be previously modified with an azide group at a specific position. This is normally achieved through enzyme-assisted incorporation of the azide moiety on the side chain of an unnatural amino acid or at a C-terminal peptide tag [34,35]. In a second step, the azide-modified protein is linked to an alkyne-modified ssDNA, either in the presence or absence of Cu(I) ions. Alternatively, the Staudinger ligation can be used to link the protein with a phosphine-modified DNA component [36].

Self-labelling protein tags. This method takes advantage of the specific activity of protein tags (previously fused to the POI) towards so-called suicide ligands. The two most popular examples are the human O6-alkylguanine-DNA-alkyltransferase (hAGT, referred to as “SNAP-tag”) [37] and the haloalkane dehalogenase (referred to as “HALO-tag”) [38]. Whereas the SNAP tag catalyzes the formation of a thioether bond between one of its Cys residues and the alkyl group of a benzylguanine-modified DNA, the HALO tag displaces the terminal chloride from a chlorohexane-modified DNA and establishes a covalent alkyl-intermediate with one of its Asp side chains. 

Expressed protein ligation. Another strategy that requires previous modification of the POI with a protein tag is the so-called “expressed protein ligation”. Here, the C-terminus of the POI is genetically fused to an intein and a chitin-binding domain (CBD) [39]. This latter serves for protein attachment to an affinity column and further intein-mediated cleavage by mercaptoethanesulfonic acid, leading to the formation of a C-terminal thioester. Under mild conditions, the thus-activated protein reacts spontaneously and selectively with the N-terminal cysteine of a DNA-peptide conjugate. The same reaction scheme can be applied to link the N-terminal of intein-fused proteins to a peptide-modified ssDNA [40]. Despite the well-defined stoichiometric composition of the final conjugate and the regioselective linkage, this method is counteracted by the insolubility of intein fusion mutants.

Enzyme-mediated ligation. A large class of covalent and regioselective DNA-protein conjugation methods is represented by enzyme-mediated ligations [46]. The variety and high-specificity of reactions offered by enzymes together with their implementation in mild aqueous conditions provide the experimenter with a wide spectrum of possibilities that can be easily applied to different scenarios. For example, the microbial transglutaminase specifically catalyzes the acyl transfer reaction between a primary amine and the γ-carboxyamide group of a Gln residue [41]. Here, the POI is genetically fused to a short peptide tag containing a Lys residue as acyl-acceptor (K-tag in Table 2) and further conjugated to a DNA-strand, previously modified at one of its termini with *N*-carbobenzyloxy glutaminyl glycine that functions as acyl-donor (Q-tag in Table 2). Another interesting strategy relies on the sortase A activity [42,43]. This enzyme catalyzes the formation of a covalent bond between two specific peptide sequences, a C-terminal LPXTG sequence and an N-terminal poly-Gly, attached respectively to one of the two components of the reaction mixture (the DNA or the POI). The two molecules are then covalently linked through a (G)_n_TXPL peptide stretch. Finally, deoxynucleotidyl transferase (TdT) has been employed to link the 3’-end of an unmodified DNA strand to a protein bearing a nucleotide triphosphate at its terminus [44]. The reaction is fast and quantitative and is particularly advantageous for label-free functionalization of multiple oligonucleotides in a one pot reaction.

DNA-templated ligation. Until now, all the methods described in this section necessitate the first step of protein engineering and an additional functionalization of the ssDNA with a specific chemical handle. This can be not only cumbersome but also extremely challenging to achieve in some cases. A novel method, recently developed by Gothelf and coworkers [45], bypasses this drawback by using a DNA/protein pair with high affinity for a metal ion and a complementary DNA strand that bears an active ester for coupling to a specific Lys-residues on the POI’s surface. In this approach, a Ni^2+^-binding tris (NTA)-modified ssDNA is bound to an His_6_-tagged protein and acts as a guide for the complementary amino-reactive oligonucleotide, which will crosslink only to the lysine residues that are close to the metal binding site. 

### 2.2. Non-Covalent Chemical Strategies

Covalent interactions are mostly strong and irreversible. When applied to the conjugation of DNA molecules and proteins, this results in a permanent chemical modification of the POI, mainly at its surface. Although useful for easier handling and manipulation purposes, the effect of such alterations on the conformational structure and enzymatic activity of the protein might be relevant and difficult to predict. In addition, the presence of a large and negatively charged DNA shell around the protein may compromise the occurrence of molecular recognition events, raising critical questions on the use of such conjugates for biological applications. The establishment and further development of non-covalent strategies for DNA-protein conjugation are, therefore, of crucial importance for the advancement of the field. 

#### 2.2.1. Non-Covalent and Non-Regioselective Methods (C-R-)

A still poorly addressed approach, which has been partially touched in recent work from us [47] and is matter of current investigation in our laboratory, relies on the use of small supramolecular ligands that display a preferential non-covalent binding to selected amino acid residues (Table 3). Previous studies have shown that guanidinio carbonyl pyrrole (GCP), and thereof derivatives, selectively bind to negatively charged Asp and Glu residues [48,49], whereas so-called molecular tweezers or clips selectively recognize the positively charged residues of Lys and Asn [50,51]. When linked to a single-stranded DNA, these non-covalent binders can be in principle used to address the surface of native proteins, avoiding prior chemical or genetic modifications. Attempts in this direction have been already initiated in our laboratory and, particularly for the tweezer strategy, have shown promising results [47]. 

The main feature of this method relies on the fact that the newly established interactions are not regioselective, meaning that all exposed amino acids of the same type will have the same probability to be targeted by the corresponding DNA-tagged ligands and their spatial discrimination on the basis of steric or local charge effects will be extremely challenging (C-R-, Figure 2c). Nevertheless, we have recently employed this apparent disadvantage to trap a single protein copy within the cavity of a large DNA origami structure (unpublished results). For this purpose, we functionalized the inner side walls of a tubular origami structure with DNA-tagged tweezers, thus allowing for the simultaneous targeting of multiple and chemically identical lysine residues exposed on the surface of the encaged protein. As our candidate protein displays a radial symmetry, this method results in the formation of a homogeneous and non-covalent DNA shell that is symmetrically distributed around a central protein oligomer. We anticipate that such a spatial configuration may offer interesting features, whose consequences on the binding efficiency and enzymatic activity of the scaffolded protein are still largely unknown. We will discuss this point more in detail in Section 3 when considering the geometry of the final DNA-protein conjugate and in the following Section 4 and Section 5, where we will examine the theoretical implications of such a topology. In principle, one might attempt to target also hydrophobic and aromatic amino acid residues of the POI. This last class of compounds, in particular, has been already employed for this purpose and includes macrocyclic systems such as cyclodextrins, cucurbiturils, and calixarenes [52,53]. These molecules form a hollow tubular structure that hosts aromatic guests in its central cavity, enabling, for example, the supramolecular association of a methylviologen-modified ssDNA to the terminal Trp side chain of the POI. 

#### 2.2.2. Non-Covalent and Regioselective Methods (C-R+)

This last class of chemical strategies combines two desirable features of DNA-protein conjugates: a non-covalent nature of their interaction and a predictable and unique positioning of the DNA strand within the final construct. Mimicking natural strategies of molecular recognition, these conjugates (and their C-R- analogs) are therefore excellent candidates to study the effect of DNA interactions on protein structure and activity. The most important examples of C-R+ methods are reported below (Table 4). We have grouped these methods according to the type of interaction taking place between the two binding partners, as suggested by Yan and coauthors in a recent review on DNA nanostructures [27]. 

Ligand-protein recognition. This is the largest category of non-covalent and regio-specific methods and includes biotin-streptavidin interaction [54], metal-induced affinity binding (typically NTA-Ni^2+^/His_6_ tag) [55], as well as the recognition of protein epitopes by DNA aptamers [56], small peptide analogs of natural substrates [47] or—in case of antibodies—by their specific antigens [57]. The successful implementation of these methods is critically dependent on the affinity of the ligand-protein interaction and the availability of synthetic or natural tags that can be appended to the DNA strand for its further conjugation to the POI.

Enzyme-cofactor reconstitution. When the enzyme needs to be stably bound to a cofactor to be active, this method demonstrated to be convenient and efficient [58,59]. Upon extraction of the cofactor from the enzyme and attainment of the corresponding inactive apoenzyme, the ssDNA is covalently linked to the cofactor and reinserted into the apoenzyme to repristinate its full activity. This method has been mainly employed for porphyrin and flavin-like cofactors and resulted in recovered activities, which were in some cases significantly different from the unmodified enzymes. Interestingly, the observed enzymatic activities appeared to be dependent on the length and content of the nucleobase sequence, raising fundamental questions on the effect of DNA tethering on protein properties.

Domain-domain interaction. A final class of C-R+ methods exploits the occurrence of DNA domains specifically recognized by DNA-binding proteins. Typical examples are zinc-finger proteins and other transcription factors [60]. 

### 2.3. From Single Stranded DNA-Protein Conjugates to Large DNA-Protein Nanostructures

The methods described above have been successfully applied for the realization of single-stranded DNA (covalently or non-covalently) linked to a POI. Such compounds, normally referred to as DNA-protein conjugates, can be further used to decorate large DNA constructs, resulting in the formation of DNA-protein nanostructures. As in most cases, the DNA-protein conjugate is not obtained as a single species, purification procedures are mandatory. The choice of the most appropriate purification procedure is, of course, dependent on the nature of the DNA-protein linkage, the stability of the protein and the type of unreacted or intermediate species present in solution.

**Figure 3:**
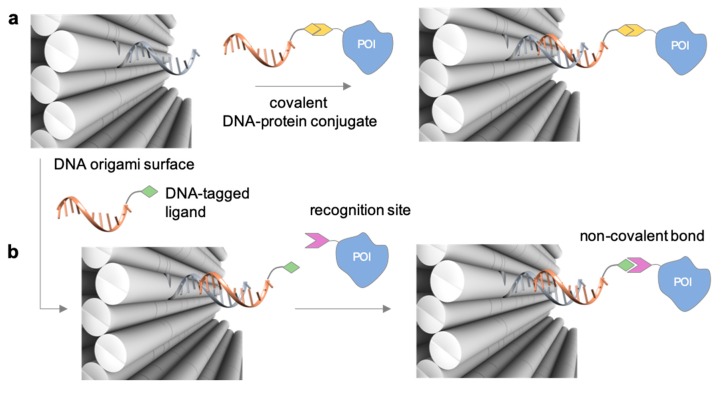
Binding strategies for the attachment of a DNA-protein conjugate to a nanostructured DNA template. The template displays a protruding arm (grey strand) to which the complementary DNA tag of the conjugate (orange strand) hybridizes. In this way, proteins can be organized in space with nanometer resolution either (**a**) through hybridization of a covalent DNA-tagged conjugate or (**b**) through non-covalent binding to a previously hybridized DNA-tagged ligand.

A detailed description of the purification procedures adopted for the isolation of DNA-protein conjugates goes beyond the purpose if this review and the interested reader may find important references to previous works in [31,61,62,63] and references therein. As a general rule, covalently linked conjugates are typically purified by chromatographic techniques, using size exclusion, ion exchange, affinity-based phases and in some cases a combination of them. Non-covalent conjugates instead are more challenging—if not even impossible—to isolate and the chance of success is highly dependent on the binding strength of the ligand. The problem becomes more difficult to solve when the DNA-protein conjugate is the result of non-regioselective methods. In those instances, the reaction products are most probably a distribution of multiple species that differ in the number of attached DNA strands and their relative location on the protein. Despite advanced analytical techniques, such as mass spectrometry [64], may enable to characterize the mixture of products, isolation of the desired DNA-protein species is still an extremely challenging task. Once the DNA-protein conjugate has been produced and isolated, the single-stranded DNA tag can be employed for anchoring the POI at the desired position of a nanostructured DNA template, obtained either by tile- or scaffold-based strategies. In principle, this final construct can be realized in two ways. In the first route, the DNA-protein conjugate (mainly covalently bound) is hybridized to the complementary strand protruding out of the DNA surface at the desired position (Figure 3a). In the second route, the two complementary strands are first hybridized at the selected position, exposing the DNA handle to which the protein will be further (normally non-covalently) linked (Figure 3b). Thus, whereas the first scaffolding strategy is driven by DNA hybridization, the second procedure relies on the binding affinity between the ligand and the protein receptor. In both cases, the final architecture will be constituted by one or several proteins tethered to a common DNA template and pre-organized in space with nanometer control over their mutual distance and relative position to the DNA walls. 

In both cases, the non-origami component is typically added in stoichiometric excess to favor the formation of the product and prevent DNA origami aggregation at high concentrations. For this reason, an accurate estimation of the concentration of the DNA-protein conjugate is at this stage not critical, particularly because the resulting DNA-protein nanostructure must be then exposed to a further purification step. The homogeneity of this final compound is indeed fundamental for unambiguous interpretation of the results obtained from binding and kinetic assays. Therefore, unreacted reagents and/or side products should be removed, isolating—when possible—a single DNA-protein nanostructure species. A series of purification strategies have been developed for the isolation of DNA-based nanoconstructs. These methods have been recently reviewed and compared in their performance by Högberg and coauthors [65], to which the interested reader should refer for more details. Briefly, the following purification procedures can be applied: ultrafiltration across membranes of defined molecular weight cut-off, gel filtration across size-exclusion resins packed within spin columns, glycerol density gradient ultracentrifugation, PEG precipitation, agarose gel extraction, fast protein liquid chromatography and separation assisted by magnetic bead capture and release of the desired probe. This latter method showed the best recovery yield and purity grade, offering a universal strategy of purification, particularly efficient for functionalized DNA origami structures. 

## 3. Geometry of the DNA-Protein Nanostructure

In this section, we will describe the spatial configuration assumed (or supposed to be assumed) by the construct composed by one or more enzymes linked to selected positions of a DNA nanostructured template, using one of the methods described above. We will distinguish three kinds of geometries, according to the shape of the template used. Conjugates organized along the growing axis of long duplexes will be considered as mono-dimensional. Complexes formed by proteins anchored at predefined positions of periodic DNA lattices or planar DNA origami structures will be instead referred to as two-dimensional. Here, one should point out that, particularly for monolayer structures, the actual shape of the DNA template is not properly planar, rather quasi-planar, due to the intrinsic flexibility of a double-helical sheet and the global curvature that accumulates when adjacent DNA duplexes are intertwined one another through crossovers. Nevertheless, as these structures basically grow along two distinct directions (one of which is the helical axis), they can be conveniently described as two-dimensional. For the same reason, protein molecules scaffolded on large DNA structures that grow along three directions will be defined as three-dimensional. A special case is represented by those constructs in which few enzymes and cofactors are linked to distinct arms of a small DNA tile. Differently from the categories described above, such nanoensembles are constituted by a single DNA unit and not by many of them linked together. Thus, despite the large flexibility of the arms enables these structures to explore a three-dimensional space, a definition of their dimensionality according to the directions of growth of the assembling units is here not appropriate. We will, therefore, treat these structures as a separate class. 

### 3.1. Mono- and Two-Dimensional Nanostructures

**Figure 4:**
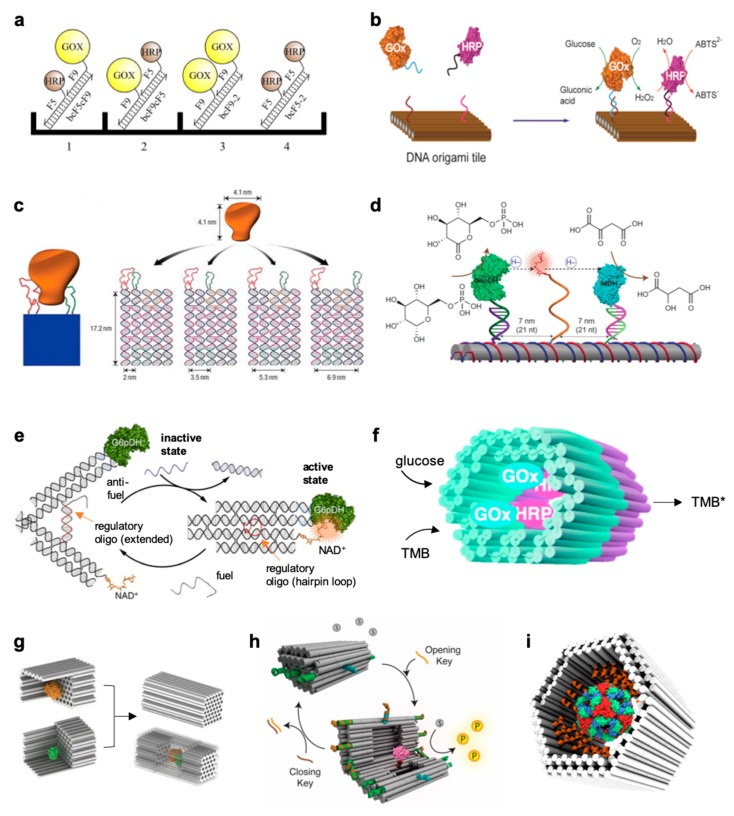
DNA-scaffolded enzymes in different geometric configurations. (**a**) Four different complexes were assembled by DNA-directed immobilization on a microplate surface, with different arrangements of GOx and HRP in homologous and heterologous pairs [66]. (**b**) Coassembly of GOx and HRP enzymes on a two-dimensional DNA origami structure with control over inter-enzyme distances [71]. (**c**) Schematic showing a rigid DNA tile (blue) that can spatially separate two ligands (red and green) at a controlled distance, with each ligand attached to a different part of the target molecule (orange) for bivalent binding [72]. (**d**) Schematic of the nanostructured complex consisting of glucose-6-phosphate dehydrogenase (G6pDH) and a malic dehydrogenase (MDH) organized on a DNA DX tile [73]. The NAD-modified single-stranded poly(T)20 is positioned halfway between the two enzymes, facilitating the transfer of hydrides. (**e**) Schematic illustration of a DNA tweezer-regulated enzyme nanoreactor [74]: a regulatory oligomer (shown in red) is designed to adopt a hairpin structure that holds the two arms of the tweezers close together, thus bringing the dehydrogenase enzyme (G6pDH) and its cofactor (NAD^+^) in close proximity (active state). The addition of a fuel strand—complementary to the regulatory loop—results in the formation of a DNA double helix between the tweezer arms that separate the enzyme/cofactor pair (inactive state). (**f**) Two separately fabricated origami units are equipped with biotinylated glucose oxidase (GOx, cyan) and horseradish peroxidase (HRP, purple), respectively, through biotin–avidin interaction [75]. The units are linked together via base-pairing, resulting in a nanoreactor that is able to perform an enzyme cascade reaction. 3,3′,5,5′-tetramethylbenzidine (TMB) is oxidized into TMB diimine (TMB*) in the presence of glucose and oxygen. (**g**) Schematic representations of the assembly of a DNA nanocage encapsulating a pair of GOx (orange) and HRP (green) enzymes [76]. Individual enzymes were first attached to half cages, followed by the addition of linker strands to combine the two halves into a full cage. (**h**) Graphic representation of the DNA vault [77], a 3D dynamic DNA nanocontainer, which encapsulates and fully encloses a protease molecule (shown in pink) in its inner cavity, thus shielding it from its substrate (gray circles) that is free in solution, and preventing formation of the product (yellow circles). The opening and closing mechanisms are triggered by the addition of specific DNA strands (in orange and brown, respectively), which operate on the lock strands of the vault (green and blue). (**i**) A hollow DNA nanocontainer has been modified in its inner cavity with peptide recognition motifs (orange helices) pre-oriented toward the binding sites exposed on the surface of the DegP protease (multicolor sphere in the middle of the container), thus leading to selective encapsulation of a single protein molecule as a consequence of multivalent binding affinity and geometric matching of the two species [47]. Adapted with permission from References [47,66,71] and [72,73,74,75,76,77] © 2017 and 2016 Macmillan Publishers Ltd and 2015 Royal Society of Chemistry.

The development of robust design strategies for the attainment of extended mono- and two-dimensional DNA frameworks greatly stimulated the interest in semisynthetic enzyme systems, with the purpose to reconstruct and manipulate multi-step cascade reactions in vitro [66,67,68,69,70,71] and multivalent binding at the surface of a single enzyme molecule [72]. Early studies of this kind have been carried out on simple mono-dimensional scaffolds [66,67] by measuring the reaction rate of enzyme pairs as a function of their intermolecular distance (Figure 4a). Using a quasi-planar surface, Yan and coauthors organized discrete glucose oxidase (GOx)/horseradish peroxidase (HRP) enzyme pairs on specific DNA origami tiles with controlled inter-enzyme spacing and position [71] (Figure 4b). By measuring the activity of the enzyme complexes at different inter-molecular distances, a dimensionally limited diffusion mechanism across connected protein surfaces could be revealed and identified as responsible for the strong enhancement in the observed reaction rate. The opposite configuration, in which distinct ligands are bound onto a DNA template and exposed to an enzyme-containing solution, has been also implemented for the systematic investigation of the bivalent binding of two distinct DNA aptamers at the opposite sides of a thrombin molecule [72]. Using atomic force microscopy, the authors could demonstrate that the efficiency of binding was dependent on the distance between the two aptamer ligands, placed on a DNA nanoarray with nanometer accuracy and used as pincers to capture and display the target protein molecule (Figure 4c). 

### 3.2. Small DNA Tiles

Whereas large mono- and two-dimensional nanostructures have been extensively used for the study of proximity effects in artificial enzyme cascades, the application of 3D templates for the same purpose has been initially limited to simple DNA junction motifs. A representative example, reported by Yan’s group in 2014 (Figure 4d), describes a two-enzyme cascade consisting of a glucose-6-phosphate dehydrogenase (G6pDH) and a malic dehydrogenase (MDH) displayed on a DNA double-crossover tile scaffold [73]. The production of NADH by G6pDH is subsequently used by MDH for reduction of oxaloacetate to malic acid. To facilitate channeling between the two enzymes, an NAD+-functionalized DNA swinging arm has been placed between the two enzymes and proven to mediate local diffusive transport through transient binding. The final construct showed not only enhanced enzymatic activity but also high specificity in a complex environment. In a different application, the distance between an enzyme and its cofactor has been varied by attaching the two components to the extremities of a DNA tweezer [74]. Upon addition of specific DNA actuators, the entire construct could be switched from an open to a closed configuration, respectively associated with an inactive and active state of the enzyme complex (Figure 4e). These and other similar examples highlight how the activity of an enzyme complex can be controlled *via* DNA, either through confinement of the reacting partners at defined positions in space or through dynamic manipulation of their range of interaction [61,78,79].

### 3.3. Large Three-Dimensional Nanostructures

Probably one of the most interesting enzyme configurations is represented by single or multiple protein molecules anchored to the inner walls of densely packed DNA origami structures. Only a few isolated examples of this kind have been reported until now [47,75,76,77] and although the observed results basically confirm what has been already found for simpler systems, the potential of these structures for in deep theoretical studies of DNA-scaffolded enzymes is undoubtedly stronger. The main features of these architectures are indeed (i) a high structural stability and (ii) an enveloping capability. Together, these properties may drastically affect the local environment near the protein surface, with important and largely still unknown consequences on its binding affinity to specific ligands and enzymatic activity towards substrates.

Using covalent DNA-protein conjugation, two distinct GOx- and HRP-loaded DNA chambers have been linked together to generate an enzyme cascade reaction inside a tubular construct (Figure 4f) [75]. Later, Yan and coworkers succeeded in fully enclosing active enzymes within a multilayered DNA nanocompartment [76], demonstrating an increased substrate turnover number for both individual enzymes and co-localized enzyme cascades (Figure 4g). In natural systems, the activity of enzymes that are fully enclosed within nanocompartments is often controlled by physically separating such enzymes from their substrates. Inspired by this generally applicable approach, Andersen and coworkers have recently developed a dynamic DNA origami nanocontainer that encapsulates single enzyme molecules and controls their catalytic activity by compartmentalization (Figure 4h) [77]. These constructs however were all assembled using DNA-enzymes conjugates obtained through covalent and non-regioselective methods raising important questions over their homogeneity and intrinsic activity prior to encapsulation within the DNA cavity. An alternative and promising method relies instead on the use of specific recognition motifs for selective and non-covalent immobilization of a single protein molecule, with control over the multivalency of the interactions and their range of action (Figure 4i) [47]. 

## 4. DNA-Nanostructures for Confined Multivalency: A Thermodynamic Model of Entropic Avidity

**Figure 5:**
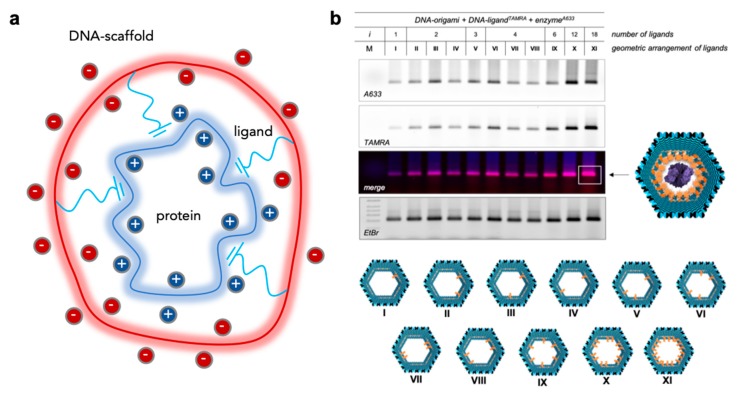
Multivalency in DNA-protein nanoconstructs. (**a**) A multivalent system composed of *n* identical ligands anchored to the same DNA scaffold and pre-oriented towards the corresponding *m* binding sites exposed on the surface of the protein receptor. The system can be analyzed with the Kitov and Bundle model enabling to extract the contribution given by the degeneracy of the system. (**b**) Agarose gel electrophoresis characterization of the non-covalent binding of a hexameric protease enzyme (DegP_6_) within the cavity of a DNA origami structure bearing distinct numbers of peptide ligands (1 *< i <* 18) in different geometric arrangements (I-XI, bottom panel). Adapted with permission from Reference [47] © 2017 Macmillan Publishers Ltd.

A special case of DNA-protein nanostructured complexes is represented by highly symmetric systems, in which a homo-oligomeric protein with *m* identical binding sites is non-covalently bound to a DNA scaffold through a number *n* of identical ligands (Figure 5a). 

For simplicity, we assume that only one ligand can bind to one receptor at a time and that all ligand/receptor binding pairs act independently and have the same binding energy. In the ideal situation in which *n* = *m*, each receptor on the protein surface is bound to its ligand partner on the scaffold and the complex is fully built. 

However, the degeneracy of the system prevents to distinguish between the different microscopic arrangements associated with the same macroscopic fully-bound state. The same concept applies to all other scenarios, in which a given number of ligands *i* < *n* will give rise to a certain number Ω_i_ of microscopic states: these are all energetically equivalent, although they are associated with the distinct ways to distribute *i* ligands among *m* available receptors. 

A very interesting model published a few years ago from Kitov and Bundle [80] provides a theoretical framework to treat these sorts of problems. Starting from the principle of additivity of binding energies [81], the model assumes that the total free energy of a macroscopic complex with *i* binding sites and degeneracy Ω_i_ is given by:

(1) $$∆G_{i}^{{}^{\circ}}=∆G_{\mathrm{inter}}^{{}^{\circ}}+\left(i-1\right)\Delta G_{\mathrm{intra}}^{{}^{\circ}}-{\mathrm{RTln}\Omega }_{i}$$

where Δ*G*_inter_ is the free energy of the initial binding event that designates the monovalent inter-molecular interaction between the first ligand and the first binding site and Δ*G*_intra_ represents instead the intra-molecular contribution given by the binding of every additional (*i* − 1) ligand to the nearby accessible binding sites of the receptor. The final term accounts for the statistical distribution of all possible microscopic species. The total energy of the macroscopic complex is therefore dependent from the energy of the individual interactions and their degeneracy. Another way to express the same concept—however in a more general and powerful manner—relies on the definition of binding avidity. If all bound species are treated collectively, we can conveniently define the avidity binding constant (*K*_avidity_) as the sum of all binding constants associated with each i-mer complex:

(2) $$K_{avidity}=\overset{i_{max}}{\underset{i=1}{\sum }}K_{i}$$

thus accounting for the cumulative effect of multivalent ligands and providing a way to quantify their global affinity. From here, the avidity binding energy can be easily calculated as:

(3) $$∆G_{avidity}^{{}^{\circ}}=-RTln\overset{i_{max}}{\underset{i=1}{\sum }}K_{i}=-RTln\overset{i_{max}}{\underset{i=1}{\sum }}e^{-∆G_{i}^{{}^{\circ}}/RT}$$

which, substituted in Equation (1) results into the following expression:

(4) $$∆G_{avidity}^{{}^{\circ}}=∆G_{inter}^{{}^{\circ}}+∆G_{intra}^{{}^{\circ}}\overset{i_{max}}{\underset{i=1}{\sum }}w_{i}\left(i-1\right)+RT\overset{i_{max}}{\underset{i=1}{\sum }}w_{i}ln\left(w_{i}/\Omega_{i}\right)$$

where *w*_i_ is the probability of the i-mer complex to occur and is defined by the Boltzmann distribution.

(5) $$w_{i}=\frac{e^{-∆G_{i}^{{}^{\circ}}/RT}}{\sum_{i=1}^{i_{max}}e^{-∆G_{i}^{{}^{\circ}}/RT}}$$

Despite its apparent complexity, Equation (4) readily captures the thermodynamic origin of multivalency and can be very useful to understand the binding properties of DNA scaffolded ligands converging to the receptor sites of a symmetric protein target. The first term of Equation (4) relates to the enthalpic contribution to multivalency given by the inter-molecular interaction between the ligand and the receptor site, which is assumed to be constant and equal for all binding sites. The second intra-molecular term is affected by the interaction between the first bound ligand and the following ones as well as by the probability of the i-mer macrocomplex to form. This value increases with the number of established bonds (*i* − 1), asymptotically approaching a maximal value. The third term describes instead the pure entropic contribution to multivalency and refers to the probability of binding rather than to its strength. This type of entropy, referred to as avidity entropy, is unique to multivalent interactions and indicates the degree of disorder in the distribution of microscopically distinct complexes. For multivalent complexes with a large number of ligands and a correspondingly large number of binding sites for protein receptor, the avidity entropy may become the major driving force for formation of the complex. Assuming that the intra-molecular term is small and equal for all microscopic complexes that describe the i-mer macrocomplex (which is indeed reasonable for large multivalent constructs), Equation (4) can be extremely simplified to

(6) $$∆G_{avidity}^{{}^{\circ}}=∆G_{inter}^{{}^{\circ}}-RTln\Omega$$

where Ω represents the total degeneracy of the system and depends only on the topology of the multivalent interactions, that is on the geometry of the ligand/protein system (a full derivation of the equations in shown in [80] for different types of topologies). 

How can we apply this model to multivalent DNA-protein complexes? What is the effect of the negatively charged scaffold on the binding energy? Preliminary studies on this largely untouched topic have been performed in our laboratory and are currently a matter of further investigation. In our previous work, a single copy of a hexameric protein (DegP_6_) has been non-covalently linked to the inner walls of a DNA origami cavity by means of a distinct number of peptide ligands that specifically address the PDZ1 domains of the protein surface (Figure 5b). Both the protein and the ligands were fluorescently labelled to enable an approximate estimation of the encapsulation yield. The results show that the binding process depends in a complex manner from the topology of the established interactions and that its efficiency grows with the number of ligands, although not in a linear fashion (Figure 5b). In other words, the global effect of multivalency cannot be expressed as the simple sum of the contributions from the single binding components. The next step will be therefore to compare the experimental observations with the theoretical expectations from the model described in Equation (6), for different ligand topologies. This will enable to quantify the effect of multivalency and in particular to reveal whether and how the negatively charged scaffold may affect the binding energy of the system. 

## 5. Catalytic Activity of DNA-Enzyme Systems: Experimental Observations and Proposed Models

**Figure 6:**
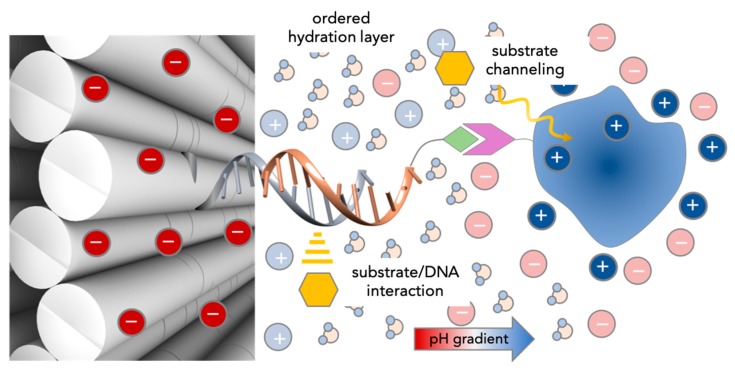
Current hypotheses on the observed increase of enzymatic activity for DNA-scaffolded enzymes. Unspecific substrate-DNA interactions can be responsible for a higher local concentration of the substrate (yellow symbol) in the vicinity of the active site. In case of enzyme cascades, the spatial proximity of the coupled enzymes facilitates the diffusion of intermediate species (substrate channeling). The highly dense and negatively charged DNA nanostructure surface and the (mostly positively) charged protein surface generate an ordered hydration layer that is supposed to favor the conformation of the protein and accelerate the reactions at its surface. Finally, the counterions at each surface are thought to establish a pH gradient, with a lower pH next to the DNA surface than in bulk solution. This is supposed to affect (mostly enhance) the catalytic activity of the enzyme.

In this section, we will try to recapitulate the knowledge acquired until now on the activity of DNA-scaffolded enzymes. Despite the diversity of experimental set ups and conditions, one common fact emerges from all studies on this topic: The reaction rate of a given enzyme or enzyme cascade is affected by the presence of DNA in solution. The extent of this effect—which may be either favorable or unfavorable—appears to be largely variable and dependent on the type of enzymes tested, the length and base sequence of the DNA strand linked (or simply mixed) to the protein and the topology of the scaffold to which the enzymes are bound. In some cases, the kinetic parameters reported by different research groups are not even in agreement, although they refer to the same enzyme system. The entire picture is therefore rather complex to grasp, probably due to the concomitant participation of several factors, whose identity and weight within the process have not yet been fully understood. In the following, we have attempted to classify the observations reported in the literature and the relative hypotheses advanced to explain them into four groups (Figure 6).

To the first class belong all those studies related to the systematic investigation of the activity of a single enzyme either in the absence or presence of DNA. Early works carried out by Niemeyer’s group evidenced the strong impact of base sequence and length on the catalytic efficiency of DNA-enzyme constructs prepared by the reconstitution of apo-myoglobin or apo-horseradish peroxidase with covalent heme-oligonucleotide conjugates [82]. In some cases, the authors observed >100-fold change in the catalytic constant. However, no clear trend could be identified, leaving the interpretation of the phenomenon still open. In another study, Wheeldon and coworkers covalently linked HRP to dsDNA fragments and tiles of different sizes [83]. Upon addition of hydrogen peroxide, the reaction rate of the modified enzymes was measured for a series of substrates that have quantifiable binding interactions with DNA, namely tetramethylbenzidine (TMB) and 4-aminophenol (4AP). The experimental results, supported by molecular simulations, revealed a mechanism of enhanced catalysis in enzyme-DNA nanostructures through increased local concentration of substrates in close proximity to the enzyme and its active site. Basically, unspecific interactions between the substrate and the DNA (i.e., not the catalyst) lead to accumulation of substrate molecules in the direct vicinity of the enzyme’s active site, explaining the higher affinity constant and rapid substrate metabolization. This phenomenon, as confirmed by Niemeyer’s previous findings, was affected by the DNA sequence and secondary structure as well as by its anchoring position on the protein surface. Interestingly, the binding of the substrate to the DNA scaffold appeared to follow a Sabatier trend [84]: weak and strong binders showed an almost negligible enhancement in kinetics, whereas intermediately bound substrates resulted in >300% increase in enzyme activity. 

The second set of data, which largely dominates the literature, is represented by enzyme cascades, mainly formed by two enzymes coupled one another directly or indirectly through a linker. The majority of these studies have been performed on the bienzyme system comprised of a GOx and an HRP. The GOx-HRP system combines two reactions: in the first step, glucose is oxidized to gluconolactone by GOx, while producing H_2_O_2_. This latter is further reduced to water by HRP with concomitant oxidation of a fluorogenic substrate such as N-Acetyl-resorufin (Ampex Red) or 2,2’-azinobis(3-ethylbenzothiazoline-6-sulfonic acid)-diammonium salt (ABTS) to, respectively, the highly fluorescent product resorufin or ABTS^+•^. Initial studies by Müller and Niemeyer in 2008 [66] explored the relationship between the activity of the bienzyme and the inter-enzyme distance by fixing GOx- and HRP-DNA conjugates on a microplate surface through DNA-directed immobilization (Figure 4a). A few years later, similar systems were independently described by Willner’s [68] and Yan’s [71] groups, and essentially confirmed the previous findings: the catalytic activity of the enzyme cascade increases for shorter inter-enzyme distances (Figure 4b). This distance-dependent or proximity effect was attributed to a facilitated diffusion process (or substrate channeling) that accelerates the transport of the product of the first enzyme, i.e., the substrate of the second enzyme, to the active site of this latter. This hypothesis was further supported by the insertion of an artificial swinging arm for rapid substrate translocation between two hydrogenase enzymes on a linear one-dimensional DNA scaffold [73] (Figure 4d). An impressive demonstration of this idea has been shown in 2016 by Yan and coworkers, through the reconstitution of an artificial multi-enzyme pathway composed of three dehydrogenase enzymes and two cofactors anchored at selected inter-molecular distances on a rectangular DNA origami platform [70]. 

Such a substrate channeling hypothesis has been later strongly debated by other researchers [85]. By connecting GOx and HRP through a small non-DNA linker, Hess and coworkers did not observe any remarkable increase in the enzymatic activity of the system, thus discrediting the proximity model. Theoretical considerations on molecular diffusion, supported by simulations, demonstrated that the activity enhancement for small substrates rapidly diffusing in solution (such as H_2_O_2_) is negligible in the time and spatial settings employed in most DNA nanotechnology reports [86]. A strong argument in favor of this point relies on the estimation of the time required by a substrate molecule to diffuse from one enzyme to the next when placed at a distance *d* in a volume *V*. According to the formula below:

(7) $$t\approx \frac{V}{4\pi \mathrm{Dd}}$$

for small volumes (0.1–10 μm^3^) and a diffusion coefficient *D* of about 1000 μm^2^/s, corresponding to a small molecule diffusing in water, a scaffold provides almost no benefit over free-floating enzymes. The observed higher throughput of scaffolded enzymes has been explained by the authors as the consequence of the establishment of a pH gradient between the DNA layer and the nearby protein surface. The low pH value experienced by the protein in the direct vicinity of the DNA scaffold is supposed to enhance the catalytic efficiency of the enzyme and in turn the performance of the full cascade. In these studies, however, the DNA structure has been modeled as an infinite planar surface with constant charge density and is—at least for a monolayer DNA origami in solution—far from a realistic situation. The formation of supramolecular aggregates, where thousands of enzyme pairs are placed in close proximity, has been also imagined as a possible source of higher turnover numbers.

Finally, the last idea has been recently postulated by Zhao et al. [76] to clarify the enhanced throughput of DNA-encaged enzymes (Figure 4g). This hypothesis relies on the formation of an ordered hydration layer between the two oppositely charged surfaces, which is supposed to stabilize the conformation of the enzyme and accelerate the reactions occurring at its surface. Although the validity of this argument has been questioned by the extremely small thickness of the water layer (few angstroms), one should fairly note that the calculated thickness of the pH gradient is also no more than 1 nm. Indeed, the “cloud” of counterions that covers a uniformly charged molecule is described by the Debye screening length (λ_D_), as follows:

(8) $$\lambda_{D}\approx \sqrt{\frac{1}{4{\pi l}_{B}C_{\mathrm{bulk}}}}$$

where *l*_B_ is the Bjerrum length (that for a molecule in water at room temperature, is about 0.7 nm) and *C*_bulk_ is the concentration of counterions in the bulk solution. This means that, for a typically charged protein in a salt solution with a *C*_bulk_ of about 100 mM, the Debye screening length is about 1 nm. In other words, beyond this distance the charge on the protein surface will not be “felt” by other charges, the electric potential will become essentially zero and the charge distribution will be uniform. Clearly, some theoretical aspects of the topic are still missing or should be reconsidered in order to be able to explain this wealth of puzzling experimental data. 

## 6. Conclusions

What we can learn from all these studies is that the properties of enzymes in the presence of DNA are far from being completely understood. In addition to the discussed results, a lot of disparities in the field can be attributed to different synthetic approaches using the same chemistry, the interpretation of successful conjugation, and the determination of DNA-protein concentration after purification—this is surprisingly difficult to accomplish and is magnified by the complexity of enzymes. Overall, better controls are required to ascertain the true enhancement of any bio-conjugated nanostructure. Indeed, most efforts until now have been focused on enzyme pairs and their putative distance-related effects. However, a major contribution to the field would ideally come from systematic studies of DNA-enzymes nanostructures containing a single copy of the protein. Using simpler constructs, the role played by the different parameters of the system can be more easily identified and quantified, starting from the molecule that links the protein of interest to the DNA scaffold. This is indeed a fundamental issue, whose importance has been often overlooked. Employing covalent and non-regioselective methods for the synthesis of DNA-enzyme conjugates (as for most of the reported works in the literature) introduces an unknown degree of variability in the activity assays, due to the intrinsic inhomogeneity of the product obtained. Even if an equimolar mixture of DNA and protein can be successfully isolated by chromatographic techniques, the final product is most probably a stochastic combination of distinct chemical species, where DNA strands are linked at different and unpredictable positions of the protein surface. Such species are extremely challenging, if not even impossible, to separate and may all contribute (however not equally) to the global kinetic properties of the conjugated- or DNA-scaffolded enzyme, thus becoming the initial source of experimental discrepancies and confusion. A model case-study would, therefore, be constituted by an enzyme that can be linked in a non-covalent fashion to the walls of a DNA origami structure, ideally in a multivalent fashion. The protein should also display an active site that is located at a position distant from the ligand recognition sites. In this way, the multivalent binding and enzymatic activity of the protein can be analyzed independently, allowing to identify and quantify the effect of DNA confinement on each of them. The further step will be then to verify whether and how these two protein features are related one to each other, for example through allosteric regulation of the protein conformation or steric issues due to different orientations of the active site with respect to the surrounding DNA walls. Surely, DNA-enzyme nanohybrid complexes will be still matter of intense studies and constructive debates, which will hopefully help us to gain a deeper insight into their interesting and partially unexpected properties. 

## Figures and Tables

**Table 1:** Representative covalent and non-regioselective (C+R-) DNA-protein conjugation methods.

C+R- (Covalent/non Regioselective Methods)		
DNA	Protein	DNA-Protein Conjugate
sulfo- SMCC crosslinking [33]		
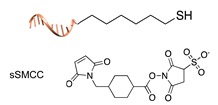	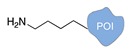	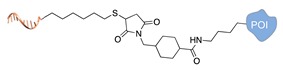
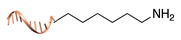	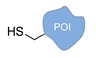	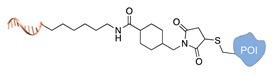

**Table 2:** Representative covalent and regioselective (C+R+) DNA-protein conjugation methods.

C+R+ (Covalent/Regioselective Methods)		
DNA	Protein	DNA-Protein Conjugate
Cu(I)-catalyzed azide-alkyne cycloaddition [34]		
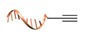	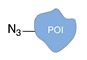	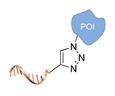
Cu(I)-free azide-alkyne cycloaddition [35]		
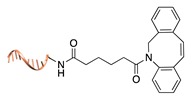	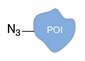	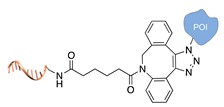
Staudinger ligation [36]		
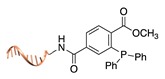	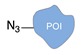	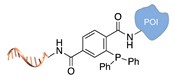
SNAP tag [37]		
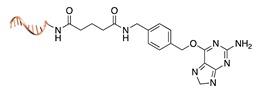	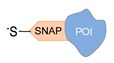	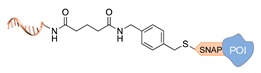
Halo-tag [38]		
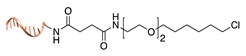	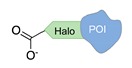	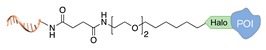
Expressed protein ligation [39,40]		
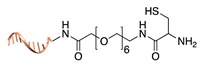	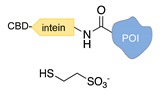	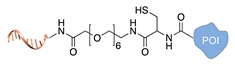
Microbial transglutaminase [41]		
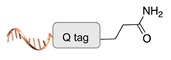	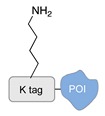	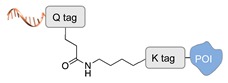
Sortase A [42,43]		
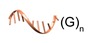	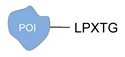	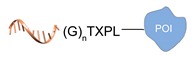
Terminal deoxynucleotidyl transferase [44]		
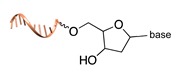	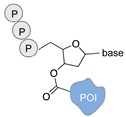	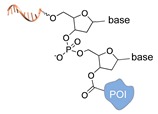
DNA-templated conjugation [45]		
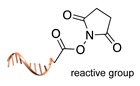	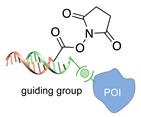	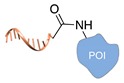

**Table 3:** Representative non-covalent and non-regioselective (C-R-) DNA-protein conjugation methods.

C-R- (Non Covalent/Non-Regioselective Methods)		
DNA	Protein	DNA-Protein Conjugate
Asp/Glu-selective GCP [48,49]		
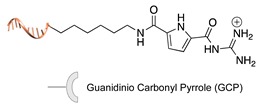	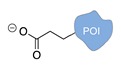	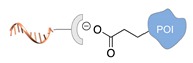
Lys/Arg-selective molecular tweezers^+^ [50,51]		
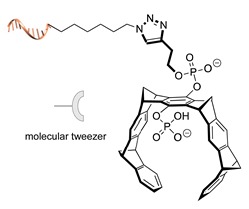	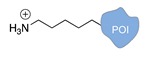	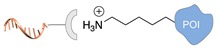
cucurbit(8)uril-mediated dimerization [52,53]		
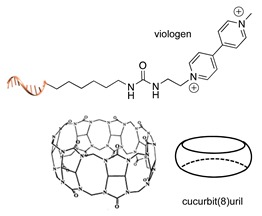	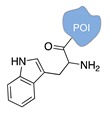	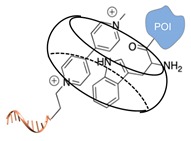

Note: The application of these methods to the synthesis of DNA-protein conjugates is still largely unexplored and is matter of current investigation in our group. The references here reported therefore refer to original works that describe the interaction between the supramolecular ligand and the POI.

**Table 4:** Representative non-covalent and regioselective (C-R+) DNA-protein conjugation methods.

C-R+ (Non Covalent/Regioselective Methods)		
DNA	Protein	DNA-Protein Conjugate
biotin/monoavidin [54]		
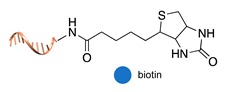	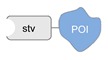	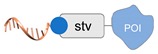
NTA/6His tag/Ni^2+^ [55]		
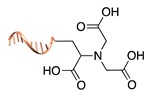	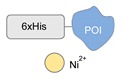	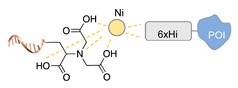
DNA aptamer/protein [56]		
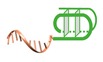	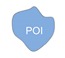	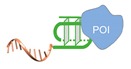
peptide ligand/protein [47]		
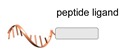	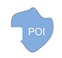	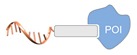
antigen/antibody-protein [57]		
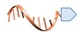	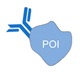	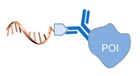
cofactor/apoenzyme [58,59]		
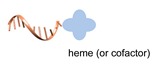	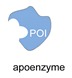	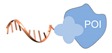
DNA binding protein [60]		
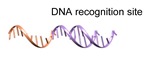	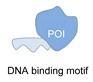